# How CBX proteins regulate normal and leukemic blood cells

**DOI:** 10.1002/1873-3468.14839

**Published:** 2024-03-01

**Authors:** Anne P. de Groot, Gerald de Haan

**Affiliations:** ^1^ European Research Institute for Biology of Ageing (ERIBA) University Medical Center Groningen (UMCG) The Netherlands; ^2^ Sanquin Research, Landsteiner Laboratory Sanquin Blood Supply Amsterdam The Netherlands; ^3^ Department of Hematology, Amsterdam UMC University of Amsterdam The Netherlands

**Keywords:** aging, CBX, epigenetics, hematopoiesis, HSC, leukemia, Polycomb, PRC1, subcellular CBX localization

## Abstract

Hematopoietic stem cell (HSC) fate decisions are dictated by epigenetic landscapes. The Polycomb Repressive Complex 1 (PRC1) represses genes that induce differentiation, thereby maintaining HSC self‐renewal. Depending on which chromobox (CBX) protein (CBX2, CBX4, CBX6, CBX7, or CBX8) is part of the PRC1 complex, HSC fate decisions differ. Here, we review how this occurs. We describe how CBX proteins dictate age‐related changes in HSCs and stimulate oncogenic HSC fate decisions, either as canonical PRC1 members or by alternative interactions, including non‐epigenetic regulation. CBX2, CBX7, and CBX8 enhance leukemia progression. To target, reprogram, and kill leukemic cells, we suggest and describe multiple therapeutic strategies to interfere with the epigenetic functions of oncogenic CBX proteins. Future studies should clarify to what extent the non‐epigenetic function of cytoplasmic CBX proteins is important for normal, aged, and leukemic blood cells.

## Graphical abstract

CBX proteins are epigenetic reader proteins that can recognize histone modifications and thereby dictate cell fate. In this review, we describe how CBX proteins dictate cell fate in normal hematopoiesis and leukemia. In addition, we discuss which CBX proteins can promote leukemic cell growth and whether pharmacological inhibition of CBX proteins can reverse leukemic cell fate.

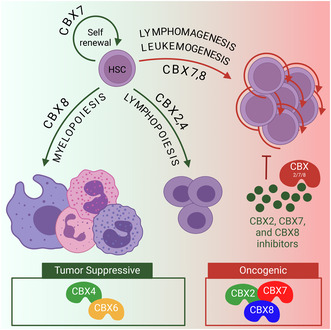

## Abbreviations

**AFF1**, acute lymphoblastic leukemia (ALL)1‐fused gene from chromosome 4

**AHD**, nuclear anchorage protein 1 (ANC1) homology domain

**AML**, acute myeloid leukemia

**ASXL**, additional sex comb like transcriptional regulator

**BCL6**, B‐cell lymphoma 6

**BCOR**, B‐cell lymphoma 6 (BCL6) corepressor

**CBX**, chromobox

**CDKN**, cyclin‐dependent kinase inhibitor

**CML**, chronic myeloid leukemia

**CtBp**, C‐terminal Binding protein

**CTL**, cytotoxic T lymphocyte

**CYP33**, cyclophilin 33

**DLBCL**, diffuse large B‐cell lymphoma

**DNMT3A**, DNA methyltransferase 3 alpha

**DOT1L**, disruptor of telomeric silencing 1‐like

**EHMT**, euchromatic histone‐lysine N‐methyltransferase

**ERG**, erythroblast transformation‐specific (ETS)‐related gene

**HDAC**, histone deacetylase

**HOXA**, homeobox A

**HSC**, hematopoietic stem cell

**HSPC**, hematopoietic stem and progenitor cell

**KD**, knockdown

**KDM2B**, lysine (K)‐specific demethylase 2B

**KMT2A**, histone‐lysine N‐methyltransferase 2A

**KO**, knockout

**LEDGF**, lens epithelium‐derived growth factor

**MDS**, myelodysplastic syndrome

**MEIS1**, myeloid ecotropic viral integration site homeobox 1

**MLLT**, mixed‐lineage leukemia translocated to

**MOF**, lysine acetyltransferase 8

**OE**, overexpression

**PAF1**, RNA polymerase II‐associated factor 1 homolog

**PcG**, Polycomb group

**PCGF**, Polycomb group ring finger

**PHC**, polyhomeotic Homolog

**PRC**, Polycomb repressive complex

**RING**, ring finger protein

**SCML**, sex comb on midleg‐like

**SETDB1**, SET domain bifurcated histone‐lysine methyltransferase 1

**SUMO**, small ubiquitin‐like modifier

**SUV39H2**, histone‐lysine N‐methyltransferase SUV39H2

**TET2**, tet methylcytosine dioxygenase 2

**TIP60**, histone acetyltransferase tat interacting protein 60 kDa

**TKI**, tyrosine kinase inhibitor

Epigenetic mechanisms regulate gene transcription and affect cell fate during development, homeostasis, regeneration, or differentiation. In this review, we focus on epigenetic regulation in hematopoietic cells. The Polycomb Group (PcG) proteins are epigenetic gene silencers that regulate hematopoietic stem cell (HSC) maintenance and, depending on which chromobox (CBX) protein is active among the PcG proteins, impose distinct HSC fates. We provide an overview on the role of CBX proteins in normal hematopoiesis, upon aging of HSCs, and in leukemia. As epigenetic mechanisms are dynamic and reversible, we suggest and describe how therapeutically targeting CBX proteins could reverse the dysregulated epigenetic landscape in leukemic cells.

## Epigenetic regulation in hematopoiesis

**Fig. 1:**
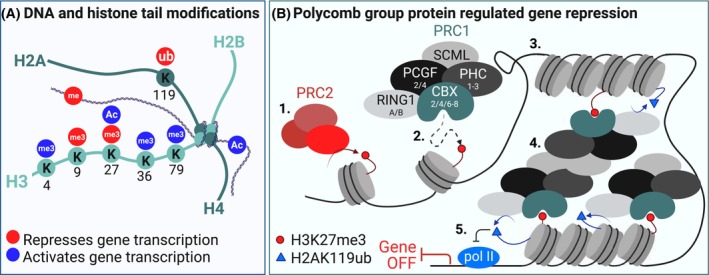
Epigenetic regulation of gene transcription. (A) DNA and histone modifications that repress or activate gene transcription. Only modification described in this review are shown. Ac, acetylation; me3, trimethylation; Ub, ubiquitination. (B) Canonical PRC2 and PRC1 dependent gene repression. (1) The PRC2 complex catalyzes trimethylation of H3K27, which can be (2) recognized by the CBX subunit of the PRC1 complex. (3) The subunit RING1 and PCGF can ubiquitinate H2AK119 which leads to chromatin compaction. (4) The subunits PHC and SCML interact with other PRC1 complexes resulting in chromatin looping and blocking (5) the transcription machinery whereby PRC targets are transcriptionally silenced. Figure created in Biorender.

Hematopoietic stem cell ensure the production of blood cells throughout life. HSCs self‐renew to maintain the stem cell pool in the bone marrow and differentiate to give rise to all the mature blood cells needed in circulation. Epigenetic regulation to control gene transcription dictates HSC fate decisions to either self‐renew or undergo differentiation. Genes important for differentiation must be epigenetically repressed to maintain the HSC pool, whereas these genes are expressed once HSCs start to differentiate. Epigenetically dictated transcription profiles are regulated by DNA and histone tail modifications that either induce or repress transcription (Fig. 1A). These modifications are recognized by epigenetic reader proteins, which can recruit multiple epigenetic writer or eraser proteins that can either add more or remove modifications. One example of a family of interacting epigenetic reader and writer proteins is comprised by the Polycomb Group (PcG) proteins.

**Fig. 2:**
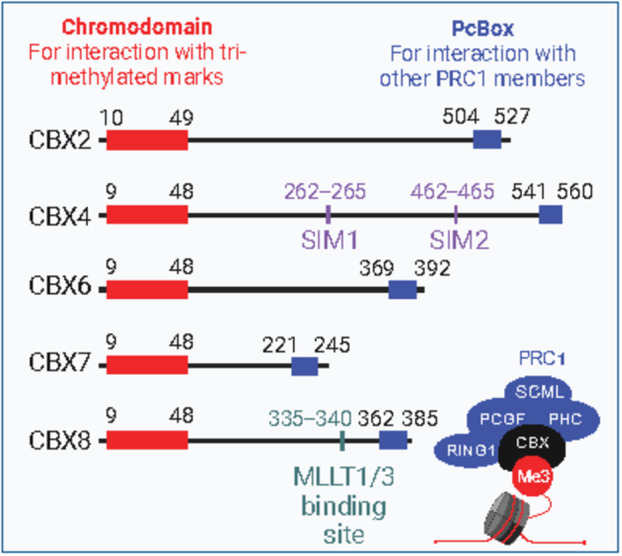
Domain annotations of CBX proteins. Each CBX protein contains a chromodomain (in red) and a PcBox (in blue). The CBX proteins use the chromodomain to interact with trimethylated marks, and the PcBox to incorporate into the PRC1 complex. CBX4 contains additional SIM motifs (purple), which are required for CBX4‐mediated SUMO E3 ligase activity. CBX8 contains a MLLT1/3 interaction site and therefore can be incorporated into the KMT2A‐r complex. The location of each feature within the human CBX protein sequence is indicated by the amino acid position above the features. Figure created in Biorender.

Polycomb group proteins are well‐known epigenetic silencers that regulate HSC self‐renewal [1, 2, 3, 4, 5, 6, 7]. The two best‐characterized multimeric PcG complexes are the Polycomb Repressive Complex 1 (PRC1) and 2 (PRC2). PRC2 initiates trimethylation of lysine 27 on histone 3 (H3K27me3), which then can be recognized by the chromodomain of one of the CBX proteins, thereby recruiting the PRC1 complex to chromatin. Subsequently, the subunits RING1 and its co‐factor PCGF initiate ubiquitination of lysine 119 on Histone 2A (H2AK119ub), resulting in local chromatin compaction. The subunits PHC and SCML can bridge multiple PcG complexes, leading to PcG complexes occupying target loci, chromatin looping, and gene silencing [8] (Fig. 1B). In Table 1, we provide an overview of current knowledge on the role of the individual PRC1 subunits in hematopoiesis. Among the various CBX proteins, only a single CBX protein (either CBX2, 4, 6, 7, or 8) can be incorporated into the PRC1 complex at any given time. CBX proteins contain a Pc‐Box domain (which ensures incorporation into the larger PRC1 complex) and a chromodomain (which recognizes chromatin modifications of histones; Fig. 2). Beyond shared genomic targets that are recognized by all CBX proteins, each CBX protein has specific target genes by which they control cell fates. Therefore, in this review, we focus on the differential effects of CBX2, 4, 6, 7, and 8 on hematopoietic cell fate. Particularly, we illustrate how some CBX proteins have oncogenic potential and promote leukemia, and suggest that pharmacological interference of the CBX proteins could reverse such changes.

**Table 1:** Canonical PRC1 subunits and their role in hematopoiesis.

Epigenetic function	Role in hematopoiesis	Role in hematopoietic malignancies
CBX2/4/6‐8		
(Reader) Recognizing H3K27me3 (CBX2/4/6‐8) or H3K9me3 (CBX2/4/7) [47, 53, 54] and thereby recruiting PRC1 to the histones; (Writer) CBX4 is a SUMO E3 ligase and SUMOylates other epigenetic proteins [14]	Cbx2 stimulates lymphopoiesis: Cbx2^−/−^ mice have small spleen and thymus and altered B‐ and T‐cell expansion and differentiation [9, 10]; Cbx2 is not required for HSC maintenance; Cbx2^−/−^ HSCs have normal repopulating capacity in primary and secondary transplantations [1]; Cbx2 stimulates hematopoietic cell differentiation: Cbx2‐OE in HSCs blocks hematopoietic proliferation and induces macrophage [1] and B‐cell differentiation [5]; CBX2 promotes the proliferation of HSPCs: CBX2‐KD reduces HSPC expansion [4]; Cbx4 stimulates hematopoietic cell differentiation: transplanting Cbx4‐OE HSCs leads to HSC exhaustion and low output of hematopoietic cells in recipient mice [5]; Cbx4 promotes T lymphopoiesis: Cbx4^−/−^ mice have small thymus, and impaired T‐cell proliferation and differentiation [11]; Cbx4 stimulates cytotoxic T lymphocyte (CTL) differentiation via SUMOylation activity: CTLs lacking the Cbx4 SUMOylation domain have a memory‐like transcriptional profile and phenotype [15]; Cbx4 is needed for neutrophil differentiation; Cbx4 inhibitors impair neutrophil development [12]; CBX6 has not yet been reported to have an obvious role in normal hematopoiesis; Cbx7/CBX7 promotes HSC self‐renewal: transplanting Cbx7/CBX7‐OE HSCs results in enhanced proliferation capacity, primary and secondary colony formation, increased primitive hematopoietic cells, and enhanced engraftment potential [5, 6]; Cbx8 is not required for normal hematopoiesis; HSPC function and hematopoietic output are not affected in Cbx8^−/−^ mice [16]; Cbx8 promotes myeloid differentiation; transplanting Cbx8‐OE HSCs leads to mature myeloid cell output in recipient mice [5]; CBX8 promotes hematopoietic progenitor expansion; CBX8‐KD HSPCs show reduced colony formation [4], whereas CBX8‐OE HSPCs show enhanced colony formation with a mild increase of primitive cells [6]	CBX2 is overexpressed in AML [29]; CBX2 promotes leukemic cell survival; CBX2‐KD reduces colony formation, and viability [29, 30], induces apoptosis [29] and myeloid differentiation [30] of leukemic cells CBX4 regulates CBX2 protein degradation via SUMOylation and thereby inhibits leukemic cell proliferation [30] CBX6 is suggested to have a tumor suppressor role in CML: CBX6 expression is low in CML, increases during TKI treatment, and CBX6 high CML patients achieve faster complete hematopoietic response than CBX6 low CML patients [31]; CBX6 is not important for hematopoietic progenitors in MDS patients; CBX6‐KD does not affect HSPCs and does not induce differentiation [32] CBX7 is upregulated in follicular lymphoma [34]; Cbx7 promotes lymphoma‐ and leukemogenesis; transplanting Cbx7‐OE HSCs results in lymphoma [34] and leukemia [5, 35] development in recipient mice, whereas Cbx7‐KO mice completely abolish the lymphoma incidents [17]; CBX7 is suggested to have a tumor suppressor role CML; CBX7 expression is low in CML, which increases during TKI treatment, is higher in the good responders, and CBX7 high CML patients achieve faster a complete hematopoietic response than the CBX7 low CML patients [31] Cbx8/CBX8 is required for KMT2A‐r leukemia development; abolishing the Cbx8‐KMT2A‐r interaction in KMT2A‐rearranged leukemias results in terminal differentiation of myeloid cells, whereas Cbx8‐KMT2A‐r binding leads to the accumulation of immature blast cells [16, 36]; CBX8 blocks erythropoiesis in CML via targeted repression of erythroid differentiation‐associated genes; CBX8‐KO CML cells show derepressed target genes and show erythroid differentiation induction [37]; CBX8 has an oncogenic role in DLBCL; CBX8‐OE promotes proliferation and blocks apoptosis in DLBCL cells [38], whereas CBX8‐KD inhibits DLBCL cell growth and induces differentiation [39]
SCMH1/L1‐2		
(Scaffold) Binding to other repressive epigenetic proteins, (e.g. DNA binding proteins [107], PRC2 [108], or PRC1 itself) supporting the spreading of PcG complexes along the chromosome and chromatin looping [109]	Scm is involved in regulating HSC activity; Scmh1 bridges PRC1 to Geminin, thereby regulating destabilizing of Geminin, resulting in reduced HSC quiescence [110].	–
PHC1‐3		
(Scaffold) Binding to other repressive proteins supporting the spreading of PcG complexes along the chromosome and chromatin looping [109, 111, 112]	Phc is required for HSC activity: Phc1^−/−^ mice have a small spleen, a mild reduction of lymphocytes, HSCs have a reduced ability to form colonies *in vitro* [110, 113, 114], and transplanting Phc1^−/−^ cells fail to reconstitute the hematopoietic system in recipient mice [113]. Phc is required for B‐cell differentiation; progenitor B cells were increased whereas mature B cells were reduced in Phc1^−/−^ mice [115]	PHC is suggested to be a tumor suppressor in infant B‐ALL: PHC1 expression is not detectable in infant B‐ALL patients with an immature B‐cell phenotype [115]
RING1A/B		
(Writer) Ubiquitination of H2AK119 [116], recruiting chromatin compaction complexes [117] or blocking RNA polymerase II activity [118] and thereby blocking transcription	Ring1 blocks hematopoietic differentiation; Ring1b^−/−^ mice have increased immature hematopoietic compartment in the bone marrow [119]; RING1 limits erythroid differentiation: RING1A inhibitors reduce colony formation and erythroid differentiation [32]	RING1A limits hematopoietic differentiation of MDS cells: RING1A‐KD induces differentiation of MDS [32]
PCGF2/4 = MEL18/BMI1		
(Writer) Cofactors of RING1 that enhances ubiquitination of H2AK119 [1, 120, 121]	Pcgf2 supports HSC maintenance: Pcgf2^−/−^ HSCs have a mild reduction in repopulating capacity [1] Pcgf4 is required for normal hematopoiesis: Pcgf4^−/−^ mice have small spleen and thymus, reduced amount of hematopoietic cells in the bone marrow, increased immature T cells, reduced mature B and myeloid cells [122, 123], reduced HSCs [123, 124, 125], and Pcgf4^−/−^ hematopoietic cells form reduced colonies *in vitro* [122, 126, 127], and reduce HSCs [128], mature myeloid [124] and lymphoid output in recipient mice [124, 129]; Pcgf4 is needed for HSC self‐renewal: Pcgf4^−/−^ HSCs have impaired growth, form less colonies, and their daughter cells rapidly lose their multilineage differentiation capacity [1]; Pcgf4 is needed for neutrophil differentiation; Pcgf4 inhibitors impair neutrophil development [12]; PCGF4 promotes HSPC self‐renewal: PCGF4‐OE HSPCs have enhanced proliferation capacity and maintain their multilineage differentiation potential *in vitro* [130]	PCGF4 is overexpressed in AML cells [131]; Pcgf4 is not needed for leukemia development; oncogenic Hoxa9‐OE Pcgf4^−/−^ cells are able to transform into AML cells [126]; Pcgf4 is needed for the maintenance of leukemic cells; Pcgf4^−/−^ HSCs have reduced proliferative capacity and they fail to repopulate recipients in secondary transplantations [126]

## The role of CBX proteins in normal hematopoiesis

To provide insight into the role of each of the various CBX proteins in the hematopoietic system, multiple studies have assessed how hematopoietic stem and progenitor cell (HSPC) were affected upon altering CBX expression levels using knockout (KO), knockdown (KD), or overexpression (OE) strategies (see Table 1).

Lymphopoiesis is altered in Cbx2‐ [9, 10] and Cbx4‐KO mice [11] shown by underdeveloped lymphoid organs and impaired lymphocyte expansion and differentiation [9, 10, 11]. This suggests that Cbx2 and Cbx4 are necessary for normal lymphopoiesis. HSCs from Cbx2‐KO mice have normal repopulating capacity [1], suggesting that Cbx2 is not required for HSC maintenance but is only needed when HSCs change cell fate toward differentiation into lymphoid lineage. In contrast, in human, CBX2‐KD reduces the proliferation and colony formation potential of hematopoietic progenitors [4]. However, in agreement with the mouse KO models, Cbx2‐OE in HSCs blocks proliferation and induces lymphoid [5], but also myeloid [1], differentiation.

Similar to Cbx2, Cbx4 also promotes differentiation of HSPCs. In line with impaired differentiation of Cbx4‐KO HSCs [11], transplantation of Cbx4‐OE HSCs leads to HSC exhaustion and reduced output of hematopoietic cells [5]. In addition, chromodomain inhibitors that prevent Cbx4 to recognize methylated histones impair neutrophil development in zebrafish [12]. CBX4 is the only CBX family member that contains SUMO interacting motifs (SIM) [13] (Fig. 2) and therefore acts as a SUMO E3 ligase [14]. Mutant Cbx4 that lacks this motif also results in impaired lymphocyte differentiation [15].

Similar as Cbx2 and Cbx4, also CBX8 promotes hematopoietic differentiation. Transplantation of Cbx8‐OE HSCs resulted in increased myeloid cell output [5]. Cbx8‐KO mice show no changes in HSPC function and hematopoietic output [16], indicating that Cbx8 is not essential for HSPC maintenance but is required when HSPCs change cell fate toward differentiation into myeloid lineage. Experiments with human cells show contradictory results, as knockdown of CBX8 in HSPCs blocks colony formation [4] and CBX8‐OE in HSPCs enhances colony formation [6], suggesting that CBX8 promotes hematopoietic progenitor expansion.

In contrast to CBX2, CBX4, and CBX8, CBX7 blocks differentiation and promotes HSPC self‐renewal. CBX7‐OE in both mouse and human HSPCs promotes proliferation and colony formation capacity, and enhances engraftment in transplantation assays [5, 6]. Furthermore, Cbx7‐KO mice do not develop lymphoma whereas Cbx7‐wild‐type mice do [17], indicating that Cbx7 has oncogenic potential and is able to push normal hematopoiesis toward lymphoma cell fate.

## Epigenetic changes upon HSC aging

Upon aging, HSCs lose their repopulation capacity and their progeny differentiates in a myeloid‐biased manner [18]. It seems plausible that the functional decline of HSCs during aging, at least in part, may be explained by a gradually changing epigenome, potentially caused by altered CBX control. Although our understanding of the complexities of the multiple epigenetic modifications that occur during HSC aging is rudimentary, several studies have provided indications that functionally relevant changes occur.

Among the CBX subunits, the expression levels of Cbx2 [19] and Cbx4 [20] have been reported to be decreased, while Cbx6 expression [21] has been shown to be increased in aged compared to young murine HSCs. As Cbx2 and Cbx4 are necessary for lymphopoiesis in normal hematopoiesis [9, 10, 11], their reduced expression levels may explain the myeloid bias observed in aging. However, we did not observe Cbx expression patterns to be consistently different in young versus aged HSCs when analyzing multiple HSC aging studies [22], indicating there is heterogeneity in aged HSC populations between mice or even between HSC clones within one mouse. In aged human HSCs, the CBX7 locus is covered with less H3K4me1 and H3K27ac levels [23], indicating reduced transcription of CBX7. Upon aging, HSCs undergo alterations in DNA methylation and the levels of the transcriptional activation marks H3K4me1 and H3K27ac levels are overall reduced [19, 23, 24, 25]. Loss of H3K27ac in aged HSCs is known to reduce the transcription of lymphoid and immune signaling genes [23]. Moreover, it has been shown that CBX2 maintains hematopoietic cells in a juvenile, lymphoid‐biased state by directly targeting and regulating ERG expression [26].

Aging often is associated with the development of age‐related clonal hematopoiesis (ARCH) or clonal hematopoiesis of indeterminate potential (CHIP), which is accompanied (and assumed to be caused) by mutations in various epigenetic modifiers including DNMT3A, TET2, and ASXL [27]. Eventually, the age‐associated epigenetic changes that result from these mutations are believed to lead to leukemia development [23, 28]. In agreement, the epigenome of aged HSCs and leukemic cells show comparable features [23].

## The role of CBX proteins in malignant hematopoiesis

The various CBX subunits have been associated with distinct functions in hematopoietic malignancies (see Table 1).

CBX2 has been shown to stimulate leukemic cell survival [29, 30]. CBX4, on the other hand, has been reported to be a leukemic suppressor, as it degrades CBX2 protein levels via SUMOylation, which leads to reduced leukemic cell proliferation [30].

Although CBX6 has not been reported to regulate hematopoietic cell fate decisions, both CBX6 and CBX7 are suggested to be tumor suppressors in chronic myeloid leukemia (CML) [31]. Tyrosine kinase inhibitor (TKI) treatment for CML patients, results in upregulation of CBX6 and CBX7 protein levels, and CML patients with low CBX6 and CBX7 levels respond better to treatment [31]. In contrast, such tumor suppressor function of CBX6 was not observed in myelodysplastic syndrome (MDS) [32].

High expression levels of CBX7 are well known to bypass senescence and extend cellular lifespan by transcriptionally repressing the CDKN2A locus, encoding the tumor suppressor protein p16^INK4A^, which is required to inhibit cell proliferation [33]. In hematopoietic cells, CBX7‐enforced self‐renewal eventually leads to lymphoma [34] or leukemia [5, 35] development.

CBX8 is specifically required for the development of KMT2A‐rearranged (KMT2A‐r) leukemia. CBX8 can interact with the KMT2A‐r complex and abolishing this interaction results in terminal differentiation of leukemic cells [16, 36]. Also, in CML, CBX8 appears oncogenic as it blocks differentiation [37]. Furthermore, enforced overexpression of CBX8 in lymphoma cells promotes proliferation and prevents apoptosis [38]. In agreement, knockdown of CBX8 in lymphoma cells results into reduced proliferation and induced differentiation potential [39], indicating that CBX8 has oncogenic potential in lymphoma as well.

In summary, in hematopoiesis, CBX2, CBX7, and CBX8 have oncogenic roles, whereas CBX4 and CBX6 function as tumor suppressors.

## Alternative CBX‐containing epigenetic complexes

**Fig. 3:**
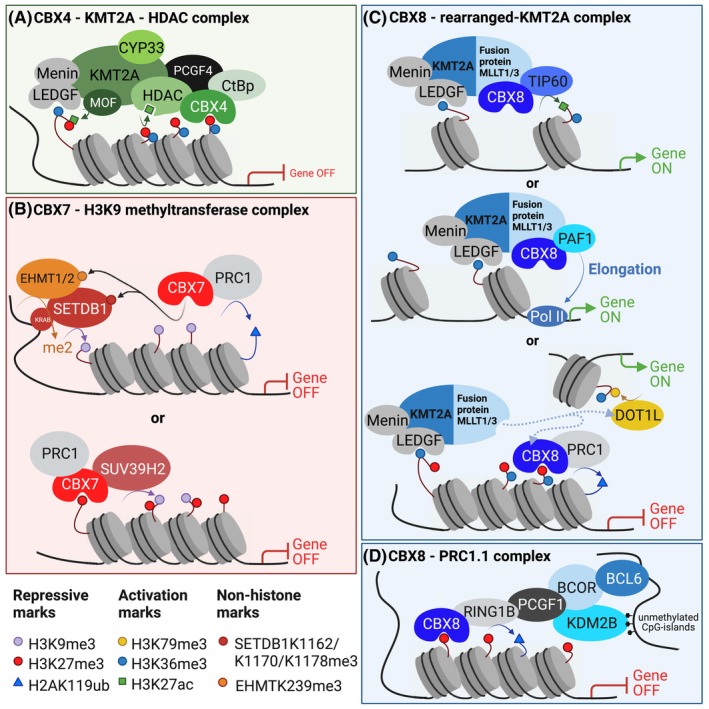
Alternative CBX‐containing epigenetic complexes. (A) CBX4, as part of the repressive complex containing HDAC, CtBp, and PCGF4, can interact with the transcriptional activator complex containing KMT2A, LEDGF, Menin, and MOF, when CYP33 is bound to KMT2A. This half‐repressive, half‐active complex can target bivalent loci by recognizing both the repressive mark H3K27me3 and the activation mark H3K36me3 by CBX4 and LEDGF, respectively. Menin is a scaffold protein that stabilizes the binding between LEDGF and KMT2A. PCGF4 and CtBp are co‐repressors and HDAC is a deacetylase that removes the MOF‐initiated acetyl groups from H3K27, which results in gene repression. (B) CBX7 can interact with the H3K9 methyltransferase SUV39H2, SETDB1, EHMT1, and EHMT2. One hypothesis how this represses genes is that CBX7 recognizes the auto‐methylated lysines on SETDB1 and EHMT and thereby recruits the CBX7‐containing PRC1 complex to H3K9me3 covered loci to repress genes in a PRC1‐dependent way. A second hypothesis is that CBX7 targets H3K27me3, thereby recruiting SUV39H2 to the chromatin which will lead to methylation of H3K9 and deeper epigenetic silencing. (C) CBX8 can interact with the KMT2A fusion partners MLLT3 and MLLT1. One mechanism to enforce transcriptional activation of rearranged KMT2A is by binding to CBX8 and TIP60 whereby TIP60‐dependent H3K27 acetylation promotes transcriptional activation. Another mechanism to promote transcriptional activation is via binding of rearranged KMT2A to CBX8 and PAF1 which then promotes transcriptional elongation. CBX8‐KMT2A‐r binding can also compete for DOTL1‐KMT2A‐r binding, thereby blocking transcriptional activation but instead initiates PRC1‐dependent gene repression at H3K36me3 active loci. (D) As part of the non‐canonical PRC1.1 complex, containing BCL6, BCOR, KDM2B, PCGF1, RING1, and CBX8, CBX8 can recruit this complex to H3K27me3 covered loci which will initiate RING1‐dependent ubiquitination of H2A and thereby repress genes. Figure created in Biorender.

Besides functioning canonically as a recruiter of PRC1, CBX proteins may also be part of alternative epigenetic complexes that regulate hematopoiesis. Four of such complexes, that we describe below, have been found in a hematopoietic context, namely, “CBX4–KMT2A–HDAC,” “CBX7–H3K9 Methyltransferases,” “CBX8–KMT2A rearranged,” and “CBX8–PRC1.1” (Fig. 3).

### CBX4–KMT2A–HDAC complex

The repressive complex containing CBX4, PCGF4, CtBp, and HDAC can interact with KMT2A [40, 41] (Fig. 3A). KMT2A, also known as mixed‐lineage leukemia (MLL), is a methyltransferase that recognizes and modifies H3K4 (H3K4me3), a mark that is associated with active transcription of KMT2A target genes, such as *HOXA9* and *MEIS1* [42, 43]. The same domain of KMT2A that recognizes H3K4me1/2 competes with binding to Cyclophilin 33 (CYP33). CYP33 binding to KMT2A increases the binding of KMT2A to the repressor proteins CBX4, PCGF4, CtBp, and HDAC. In this process, KMT2A‐mediated H3K4 methylation is blocked, whereas HDAC‐mediated H3K27 deacetylation increases, thereby allowing KMT2A to switch from an activator to a transcriptional repressor [40, 41]. In leukemia, the *KMT2A* gene is often translocated and fused to *AFF1* (also known as *AF4*), *MLLT1* (also known as *ENL*), or *MLLT3* (also known as *AF9*) [44]. This results in a rearranged KMT2A (KMT2A‐r) protein that lacks the CYP33 binding domain [44, 45], thus abolishing the negative feedback regulation of KMT2A‐regulated transcription and continuous activation of KMT2A target genes [45], which then drives leukemogenesis. The interaction between CBX4, HDAC, and KMT2A therefore seems to prevent leukemogenesis.

Besides its function as an epigenetic reader, HDAC inhibitors stimulate the enzymatic writer function of CBX4, thereby promoting CBX4‐regulated SUMOylation and degradation of CBX2, resulting in impaired leukemic cell growth [30].

### CBX7–H3K9 methyltransferase complex

Beyond canonical binding to H3K27me3, CBX7 has been reported to also bind the H3K9 methyltransferases SUV39H2 [46], SETDB1 [6, 47, 48], EHMT1, and EHMT2 [6], and thereby, CBX7 can regulate gene repression in H3K9me3‐covered loci (Fig. 3B). This interaction requires these histone methyltransferases to undergo auto‐methylation to mimic the trimethylated histone modifications, which all four methyltransferases are able to do [47, 49, 50, 51, 52]. However, SUV39H2 has been reported to interact with CBX7 regardless of auto‐methylation. It has been shown that SUV39H2 normally does not target the CDKN2A locus, which encodes for the tumor suppressor protein p16^INK4A^. However, enforced expression of CBX7 enhances binding to SUV39H2 and recruits SUV39H2 to this locus resulting in H3K9me3 modification [46]. Therefore, CBX7 is thought to promote deeper chromatin compaction to stably silence target genes. We have previously shown that in a hematopoietic context, SETDB1, EHMT1, and EHMT2 are trimethylated and that they can interact with CBX7. We also showed that CBX7 is recruited to H3K9me3‐covered loci [6], and therefore, we suggested that SETDB1, EHMT1, and EHMT2 target H3K9‐methylated loci and recruit CBX7 to these loci. Note that CBX7 can also directly bind H3K9me3 with its chromodomain [47, 53, 54].

### CBX8–KMT2A‐r complex

Besides the canonical role of CBX8 as a repressor in the PRC1 complex, CBX8 can also act as a transcriptional activator (Fig. 3C). Similar to CBX4, CBX8 can interact with the KMT2A complex, however, only when KMT2A is fused to one of its fusion partners MLLT1 [36, 39, 55, 56, 57, 58, 59] or MLLT3 [16, 39, 57, 58, 60, 61, 62, 63, 64]. CBX8 can recognize the AHD domain [63], which is present in both MLLT1 and MLLT3 (Fig. 2) but not in other KMT2A fusion partners. The fusion partners MLLT1 and MLLT3 are responsible for sustained expression of HOXA9 and MEIS1, which then drives KMT2A‐r leukemic cell proliferation.

When bound to the MLLT1‐KMT2A fusion protein, CBX8 cannot initiate PRC1‐dependent ubiquitination of H2Aub and there is less H3K27me3 marks at the *HOXA9* and *MEIS1* loci [36]. CBX8‐MLLT1‐KMT2A‐r instead promotes PAF1‐dependent transcriptional activation [65] resulting in leukemic transformation [36, 65] (Fig. 3C). Similar to MLLT1 binding, when bound to the MLLT3‐KTM2A‐r fusion protein, CBX8 stimulates *HOXA9* gene transcription by recruiting the histone acetyltransferase TIP60 to the *HOXA9* locus, also resulting in leukemic transformation [16] (Fig. 3C).

In contrast, others suggest that the CBX8–MLLT3–KTM2A‐r complex reduces H3K79me3 levels [59] and induces transcriptional repression of *HOXA9* and MEIS1 accompanied with differentiation and reduced self‐renewal of HSPCs [58, 59]. CBX8 is able to simultaneously bind MLLT3 and the PRC1 subunit RING1 [60], and therefore, CBX8 may recruit the repressive PRC1 complex to active KMT2A‐target loci and switch those loci, via PRC1, to a repressive state. Moreover, MLLT3 and MLLT1 mutually exclusively bind either CBX8 or the H3K79me3 methyltransferase DOT1L, and thereby, CBX8‐MLLT3 or CBX8‐MLLT1 binding may block the transcriptional activation role of KTM2A‐r [58, 59, 63] (Fig. 3C). In contrast, the replacement of DOT1L by CBX8 reduced DOT1L‐mediated H3K79me3, leading to transcriptional activation instead of repression [62]. This suggests that DOT1L may compete with CBX8 in regulating gene activation in a TIP60 or PAF1 context, instead of competition between DOT1L and CBX8‐PRC1 binding.

Although it remains unclear whether CBX8 acts as a transcriptional activator or repressor, knockdown studies point out the importance of CBX8 in KMT2A‐r leukemia. In leukemic cells, downregulation of CBX8 led to reduced HOXA9 expression and reduced cell growth in KMT2A‐r but not in wild‐type KMT2A leukemic cells [66]. However, others found that downregulation of CBX8 affects cell growth of HSPCs with wild‐type KMT2A [4] but not of rearranged KMT2A‐transduced HSPCs [67]. However, in both conditions, downregulation led to reduced HSPC colony formation [4, 67]. Interestingly, blocking the chromodomain of CBX8 in leukemic cells using a small molecule, reduced both CBX8 and MLLT3 binding to the *HOXA9* locus, whereby HOXA9 levels were repressed, indicating that CBX8‐MLLT3‐KMT2A‐r functions as a transcriptional activator. Moreover, inhibition of CBX8 reduced cell growth of KMT2A‐r leukemic cells but not of wild‐type KMT2A leukemic cells [66]. In addition, CBX8 is not required for normal hematopoiesis, as deletion of CBX8 did not affect HSPCs function [16]. Therefore, CBX8 inhibitors may be used to target KMT2A‐r leukemic cells.

### CBX8–PRC1.1 complex

CBX8 has also been suggested to regulate gene repression as part of the non‐canonical PRC1.1 complex (Fig. 3D). It was shown that BCOR can interact with CBX8, BCL6, and with the PRC1.1 members, PCGF1, RING1, and KDM2B [39]. Moreover, CBX8 interacted with BCOR in diffuse large B‐cell lymphoma (DLBCL) cells. Downregulation of CBX8 blocked BCOR recruitment and H2A ubiquitination formation at bivalent H3K27me3 loci accompanied with reduced cell growth of DLBCL cells [39]. The PRC1.1 complex is important for leukemic cell growth as well [67]. In leukemic cells, the PRC1.1 complex is recruited to target loci in an H3K27me3‐independent manner, as both PCGF1 and KDM2B are found at these loci [67], and KDM2B is known to directly recruit PRC1.1 to DNA by binding non‐methylated CpG islands [68, 69]. Furthermore, knockdown of the PRC1.1 members PCGF1, RING1, KDM2B, and BCOR reduced leukemic cell growth. Although CBX8 was found as a PCGF1 interactor in myeloid leukemia cells [67], molecular evidence of CBX8 as being an active member in the PRC1.1 complex in leukemic cells is lacking.

## Pharmacologically targeting the CBX proteins as a novel anti‐leukemic therapy

Currently, multiple CBX‐chromodomain inhibitors exist, such as the CBX2 inhibitor SW2_152F [70], the CBX7 inhibitors MS452 [48] and MS351 [71], the CBX8 inhibitors SW2_110A [66] and UNC7040 [72], and the dual‐targeting CBX4/7 inhibitors UNC3866 [73], and UNC4976 [74]. All these inhibitors interact with the chromodomain and prevent CBX binding to trimethylated histone marks thus preventing gene repression.

Pharmacological targeting of CBX proteins may be an effective strategy to eliminate leukemic cells. However, inhibiting CBX proteins as a therapeutic option in a hematopoietic context should be taken carefully as these proteins have both oncogenic and tumor suppressor functions. For example, CBX4 [30] and CBX6 [31] are tumor suppressors in hematopoietic cells and therefore do not qualify as drug targets in leukemia. In contrast, CBX2 [29, 30], CBX7 [5, 6, 17, 34, 35], and CBX8 [16, 36, 37, 38, 39] have oncogenic potential in hematopoiesis as they promote the survival of leukemia [5, 6, 16, 29, 30, 35, 36, 37] and lymphoma [17, 38, 39] cells. Therefore, pharmacologically targeting the oncogenic CBX proteins, CBX2, CBX7, and CBX8 could be a promising therapeutic strategy to epigenetically reprogram and eliminate leukemic cells. Table 2 summarizes the various therapeutic approaches to interfere with these oncogenic CBX complexes resulting in anti‐leukemic outcomes.

**Table 2:** Drug‐targeting epigenetic proteins in hematological malignancies. ALL, acute lymphoblastic leukemia; AML, acute myeloid leukemia; CALM, clathrin assembly lymphoid myeloid; CLL, chronic lymphocytic leukemia; CML, chronic myeloid leukemia; CRC, colorectal cancer; DLBCL, diffuse large bB‐ cell lymphoma; KMT2A‐r, KMT2A‐rearranged; MM, multiple myeloma; NPM, nucleophosmin; NSCLC, non‐ small‐ cell lung cancer; PDAC, pancreatic cancer; SLL, small lymphocytic lymphoma.

Drug‐target	Available inhibitors	Preclinical hematopoietic studies	Clinical trials
The canonical CBX2, CBX7 and CBX8 containing complexes			
CBX2	SW2_152F [70]	–	–
CBX7	UNC3866 [73] UNC4976 [74] MS452 [48] MS351 [71]	MS452 reduces cell growth and induces differentiation of leukemic cell lines [6]. MS452 reduces primary AML cell growth [6]	–
CBX8	SW2_110A [66] UNC7040 [72]	SW2_110A reduces cell growth of KMT2A‐r leukemic cell lines [66] and lymphoma cell lines [72]	–
PCGF4 (=BMI1)	PTC209 [132] PTC596 [133] QW24 [134] PRT4165 [135]	PTC209 induces apoptosis and reduces viability of T‐lymphoblastic [136] and AML cell lines [136, 137], and primary CALM‐AF10 AML cells [138]. PTC209 blocks clonogenic activity of CML cells [139] PTC596 induces apoptosis and reduces viability in AML cell lines [133, 138, 140] and primary AML cells [133]. PTC596 reduces AML cell line engraftment in immune deficient mice which prolongs mouse survival [133, 138] PRT4165 reduces T‐lymphoblastic and AML cell line viability [136]	PTC596 is in Phase 2 trial for Leiomyosarcoma patients, NCT03761095
RING1	RB‐3 [141] PRT4165 [135]	–	–
The alternative CBX7 – H3K9 methyltransferase complex			
SUV39H2	OTS193320 [142]	–	–
SETDB1	Emetine [143] APQ [144] SETDB1‐TTD‐IN‐1 [145]	Emetine reduces ALL [75, 76] and AML [77] cell line viability via apoptosis induction. Emetine reduces viability and colony formation of primary AML cells [77]. Emetine reduces viability and induces apoptosis of primary CLL cells [78]. Emetine reduces engraftment of murine CLL and prolongs mouse survival [78]. Emetine reduces AML cell line engraftment in immune deficient mice [77] SETDB1‐TTD‐IN‐1 affects gene expression in KMT2A‐r AML cell lines [145]	Emetine is in Phase2/3 trial for COVID‐19 patients, NCT05889793
EHMT1/EHMT2	BIX01294 [146] UNC0224 [147] UNC0321 [147] UNC0631 [148] UNC0646 [148] UNC0638 [149] UNC0642 [150] A366 [151] SDS‐347 [152]	BIX01294 inhibits proliferation and induces apoptosis of T‐ALL [79], DLBCL [153], and AML [80, 81, 82, 83] cell lines, however, BIX01294 had less effect in the specific AML cell lines K562, KG1, and Kasumi [81, 82]. BIX01294 induces differentiation of AML cell lines [80, 82, 83] UNC0646 reduces cell viability of T‐ALL [84] and B‐CLL [85] cell lines. UNC0646 induces apoptosis in T‐ALL cell lines [84] UNC0638 reduces cell viability of T‐ALL [86] cell lines, primary AML cells [87], and leukemic murine cells [87, 88]. UNC0638 induces differentiation of primary AML cells and leukemic murine cells [87] UNC0642 reduces cell viability of T‐ALL [86] and CML [89] cell lines. UNC0642 induces apoptosis and reduced colony formation of CML cell lines and primary CML cells [89]. UNC0642 reduces T‐ALL cell line growth in xenograft mice [86]. UNC0642 reduces leukemic cell growth in a murine CML mouse model and a human CML xenograft model [89] A‐366 reduces cell growth of T‐ALL and AML cell lines [90]. A‐366 induces differentiation of AML cell lines [90]. A‐366 reduces AML cell line growth in xenograft mice [90]	
The CBX alternative CBX8 – KMT2A‐r complex			
Menin	There are five clinical trials for leukemia patients, and two trials for non‐leukemia patients; KO‐539 (Ziftomenib) is in phase 1 trial for relapse and refractory AML patients and in phase 2 trial for NPM1‐mutated AML patients, NCT04067336; DS‐1594b is in phase1/2 trial for AML and ALL patients, NCT04752163; JNJ‐75276617 is in Phase 1 trial for AML and ALL patients, NCT04811560; BMF‐219 is in Phase 1 trial for AML and ALL with a translocated *KMT2A* gene or NPM1 mutations, DLBCL, MM, CLL and SLL patients, NCT05153330; BMF‐219 is in Phase1/2 trial for Type2 Diabetes patients, NCT05731544; BMF‐219 is in Phase1/1b trial for NSCLC, PDAC and CRC patients, NCT05631574; SNDX‐5613 is in Phase1/2 trial for AML patients, NCT05360160		
TIP60	NU9056 [154] MG149 [155] TH1834 [156]	NU9056 reduces proliferation via apoptosis in NK/T lymphoma cells [91]	‐
The CBX8 – PRC1.1 complex			
BCL6	79‐6 [157] FX1 [93] BI‐3812 [158] BCL6‐i [159] TMX‐2164 [160] Ap‐4‐287 [161] GSK137 [162] CCT369347 [163] CCT372064 [164] WK500B [94] OICR12694 [95]	79‐6 blocks proliferation of ALL cell lines [96]. 79‐6 reduces proliferation and induces apoptosis of lymphoma cells in a cell line xenograft mouse model [93] FX1 reduces cell growth of lymphoma cell lines [93]. FX1 reduces proliferation and induces apoptosis of lymphoma cells in a cell line xenograft mouse model [93, 94] WK500B reduces cell growth and induces apoptosis of DLBCL cell lines [94]. WK500B reduces the proliferation of lymphoma cells in a cell line xenograft mouse model [94] OICR12694 (JNJ‐65234637) blocks DLBCL cell line growth [95]	–

When directly targeting the CBX subunits, we have previously shown reduced leukemic cell growth and induction of differentiation after treatment with the CBX7 inhibitor MS452 [6]. Inhibition of CBX8 with UNC7040 prevents binding of CBX8 to H3K27me3‐covered loci, which leads to reduced cell growth of lymphoma cells [72]. In addition, as discussed earlier, inhibiting CBX8 with SW2_110A reduced leukemic cell growth as well [66]. This indicates that both CBX7 and CBX8 are candidate therapeutic targets to eliminate leukemic cells. Although PCGF4 and RING1 inhibitors also exist and anti‐leukemic effects have been observed using these inhibitors (Table 2), pharmacological inhibition of PCGF4 and RING1 may result in unwanted effects as these proteins can also partner with the tumor suppressor CBX proteins CBX4 and CBX6 to form the PRC1 complex.

Besides targeting the canonical CBX‐containing PRC1 complex, pharmacologically targeting the subunits of the alternative complexes could also be a strategy to eliminate leukemic cells. Non‐canonical CBX7‐regulated gene repression may be blocked by pharmacologically inhibiting SETDB1, EHMT1, EHMT2, or SUV39H2 and thereby preventing recruitment of CBX7 to chromatin, or preventing H3K9me3‐mediated chromatin compaction. Indeed, anti‐leukemic effects were observed after inhibiting SETDB1 [75, 76, 77, 78], EHMT1, and EHMT2 [79, 80, 81, 82, 83, 84, 85, 86, 87, 88, 89, 90]. However, additional studies are needed to understand whether CBX7 function is affected by these inhibitors.

PRC1‐independent CBX8 repression may be blocked by inhibiting Menin, which will disrupt the interaction between Menin and KMT2A‐r, thus preventing the recruitment of the CBX8‐KMT2A‐r complex to chromatin. Indeed, Menin inhibitors turn out to be promising drugs to treat leukemia. Extensive pre‐clinical work has been done to develop and test Menin inhibitors for leukemia treatment (not described here), which led to multiple clinical trials (listed in Table 2). To block the consequences of the PRC1‐independent CBX8 complex, gene transcription may be blocked by preventing H3K27 acetylation using TIP60 inhibitors. Although anti‐leukemic effects using such inhibitors have not been reported, TIP60 inhibition results in induced apoptosis and reduced proliferation of lymphoma cells [91].

To block CBX8‐PRC1.1 activity in leukemia, BCL6 inhibitors may be used. BCL6 binds DNA directly, and therefore, inhibition of BCL6 may block the recruitment of the CBX8‐PRC1.1 complex to BCL6‐specific targets. As reviewed before [92], the PRC1.1 complex containing both BCL6 and CBX8 may be tissue‐specific and restricted to the lymphoid lineage only. Therefore, inhibition of BCL6 could be a good strategy to block CBX8‐PRC1.1 function in lymphoid malignancies. Indeed, several studies show that BCL6 inhibitors reduce cell growth of lymphoma [93, 94, 95] and lymphoid leukemic [96] cells.

Taken together, anti‐leukemic effects can be achieved by direct drug‐targeting CBX proteins, but also by pharmacological targeting subunits of alternative CBX‐containing complexes.

## Non‐epigenetic role of CBX proteins in cancer

As described above, CBX proteins exert their function in the nucleus as epigenetic regulators. However, multiple studies have reported that in cancer cells CBX proteins primarily localize in the cytoplasm [97, 98, 99, 100, 101, 102, 103]. For example, cytoplasmic CBX2 is associated with progression of breast cancer [101] and prostate cancer [100]. Cytoplasmic CBX4 has been correlated with enhanced hepatocellular carcinoma cell growth, metastasis ability, and reduced survival of hepatocellular carcinoma patients [102]. It has been proposed that CBX4 is retained in the cytoplasm through interaction with the glutamate metabotropic receptor 4 (GRM4), which then blocks nuclear CBX4‐mediated gene regulation, proliferation, migration, and invasion ability of osteosarcoma cells [103]. Cytoplasmic localization of CBX6 and CBX8 has not been described so far. CBX7 is known to have a dual role in cancer cell proliferation. In some cancers, CBX7 promotes proliferation, whereas in other CBX7 inhibits cell growth (reviewed by Li *et al*. [104]). Interestingly, two CBX7 isoforms exist, of which the long isoform p36^CBX7^ is localized in the nucleus, while the short p22^CBX7^ isoform is cytoplasmic. Nuclear p36^CBX7^ is the primarily expressed isoform in proliferating cells and, as expected, promotes proliferation. Serum starvation blocks cell proliferation and induces the expression of the cytoplasmic p22^CBX7^ isoform. Indeed, when overexpressed, cytoplasmic p22^CBX7^ blocks proliferation [105]. In cardiac cells, p22^CBX7^ is the main isoform and has been demonstrated to interact with cytoplasmic proteins that stabilize cell cycle arrest transcripts like CDKN1A. Thereby, cytoplasmic CBX7 blocks proliferation of cells [106].

## Conclusions and future perspectives

CBX proteins control the fate of HSCs. While CBX7 regulates their self‐renewal, CBX2, CBX4, and CBX8 induce lineage commitment. CBX2, CBX7, and CBX8 have oncogenic activity in hematopoiesis. Therefore, future studies may consider elaborating on the use of CBX2, CBX7, and CBX8 inhibitors as leukemia treatment. However, these treatment strategies should be considered carefully as it remains unclear how (and when) the CBX proteins regulate both normal and malignant hematopoiesis.

Besides functioning in the nucleus as epigenetic regulators, CBX proteins may also promote cancer progression via cytoplasmic mechanisms. Future studies should clarify whether, similar to other cancer cell types, cytoplasmic CBX2 and CBX4 induce leukemia, whereas cytoplasmic CBX7 blocks leukemia progression and if so, whether specifically targeting either nuclear or cytoplasmic CBX proteins to inhibit leukemia progression is warranted. In addition, future research should investigate whether the non‐epigenetic role of the CBX proteins differs between normal, aged, and leukemic blood cells, and if so, whether targeting aged and (pre‐)malignant stem cells is feasible, while at the same time sparing young‐like and healthy blood cells.
